# Prediction of ipsilateral lateral cervical lymph node metastasis in papillary thyroid carcinoma: a combined dual-energy CT and thyroid function indicators study

**DOI:** 10.1186/s12885-021-07951-0

**Published:** 2021-03-04

**Authors:** Ying Zou, Huanlei Zhang, Wenfei Li, Yu Guo, Fang Sun, Yan Shi, Yan Gong, Xiudi Lu, Wei Wang, Shuang Xia

**Affiliations:** 1 Department of Radiology, First Teaching Hospital of Tianjin University of Traditional Chinese Medicine, No. 314 Anshan West Road, Nan Kai District, Tianjin, 300193 China; 2 Department of Radiology, First Central Clinical College, Tianjin Medical University, No. 24 Fu Kang Road, Nan Kai District, Tianjin, 300192 China; 3 Department of Radiology, Yidu central hospital of Weifang, No. 4138 Linglongshan nan Road, Qing Zhou City, Shandong, 262500 China; 4 Department of Radiology, The First Hospital of Qinhuangdao, No.258 Wenhua Road, Haigang District, Qinhuangdao, 066000 China; 5 Department of Radiology, Tianjin First Central Hospital, School of Medicine, Nankai University, No. 24 Fu Kang Road, Nan Kai District, Tianjin, 300192 China; 6 Department of Ultrasonography, Binzhou Medical University Hospital, No. 661 Huanghe 2nd Road, Binzhou City, 256603 Shandong China; 7 Department of Radiology, Tianjin Hospital of ITCWM Nan Kai Hospital, No.6 Changjiang Road, Nan Kai District, Tianjin, 300100 China; 8 Department of Otorhinolaryngology, Tianjin First Central Hospital, No. 24 Fu Kang Road, Nan Kai District, Tianjin, 300192 China

**Keywords:** Papillary thyroid carcinoma, Lateral cervical lymph node metastasis, Dual-energy computed tomography, Iodine concentration, Tg, Anti-Tg, Extrathyroidal extension

## Abstract

**Background:**

Predicting the possibility of ipsilateral lateral cervical lymph node metastasis (ipsi-LLNM) was crucial to the operation plan for patients with papillary thyroid carcinoma (PTC). This study aimed to investigate the independent risk factors for ipsi-LLNM in PTC patients by combining dual-energy computed tomography (DECT) with thyroid function indicators.

**Methods:**

We retrospectively enrolled 406 patients with a pathological diagnosis of PTC from Jan 2016 to Dec 2019. Ensure the DECT images were clear and the thyroid function indicators were complete. Univariate and multivariate logistic analyses explored the independent risk factors for ipsi-LLNM. To evaluate the cutoff value of each risk factor by using receiver operating characteristic (ROC) curves.

**Results:**

A total of 406 patients with PTC were analyzed, including 128 with ipsi-LLNM and 278 without ipsi-LLNM. There were statistical differences of parameters between the two groups (*P* < .0001), including serum Tg, Anti-Tg, Anti-TPO, the volume of the primary lesion, calcification, extrathyroidal extension (ETE), and iodine concentration (IC) in the arterial and the venous phases. Independent risk factors for ipsi-LLNM included serum Tg, Anti-Tg, ETE, and IC in the arterial and the venous phases (*P* < .05). The combined application of the above independent risk factors can predict the possibility of ipsi-LLNM, with an AUC of 0.834. Ipsi-LLNM was more likely to occur when the following conditions were met: with ETE, Tg >  100.01 ng/mL, Anti-Tg >  89.43 IU/mL, IC in arterial phase > 3.4 mg/mL and IC in venous phase > 3.1 mg/mL.

**Conclusions:**

The combined application of DECT quantitative parameters and thyroid function indicators can help clinicians accurately predict ipsi-LLNM before surgery, thereby assisting the individualized formulation of surgical procedures.

**Supplementary Information:**

The online version contains supplementary material available at 10.1186/s12885-021-07951-0.

## Background

The incidence of papillary thyroid carcinoma (PTC) has dramatically increased during recent years [1], and it is well established that PTC has a strong propensity for lymph node metastasis (LNM) [2], which may increase the recurrence and shorten survival [3, 4]. According to the American Thyroid Association (ATA) management guidelines for adult patients with thyroid cancer [5], patients with N1 stage (presence of regional LNM) should perform lateral cervical lymph node dissection (LLND). However, prophylactic LLND for low-risk patients (e.g., no clinical or radiographic evidence of invasion or metastases) [5] will undoubtedly increase the probability of postoperative complications. Therefore, it is critical to predicting lateral cervical LNM (LLNM) as accurately as possible before surgery.

Preoperative imaging examination plays an essential role in the detection and staging of LLNM in patients with PTC [6]. However, ultrasound (US), which is the first-choice examination method for thyroid cancer [7], has high specificity but low sensitivity for lateral cervical lymph node examination [8–10]. Therefore, for most lymph nodes without typical image characteristics, US examination is considered insufficient. Moreover, US is greatly affected by the operators’ experience and manipulation [11], and cannot achieve quantitative measurement. Dual-energy computed tomography (DECT) is widely used to help differentiate metastatic from benign lymph nodes in patients with PTC in recent years [12–17]. Both the 2018 and 2020 versions of the National Comprehensive Cancer Network (NCCN) guidelines [18, 19] clearly stated that the possible potential delay in postoperative radioactive iodine (RAI) therapy caused by the use of iodinated contrast agents would not cause harm to PTC patients. Previous studies have shown that the combination of the slope of the energy spectrum curve (λ_HU_) in the venous phase and normalized iodine concentration (NIC) in the arterial phase showed higher accuracy for the preoperative diagnosis of LNM [17]. But the measurement object was a lymph node, which was complicated and hard to achieve a one-to-one correspondence between DECT and pathology in clinical work. In addition, DECT-based radiomic nomogram improved the preoperative prediction of LNM in patients with PTC, and the area under the receiver operating characteristic (ROC) curve (AUC) was 0.807 to 0.910 in the training cohort [14, 20]. However, the above studies have focused on LNM, not just LLNM. Since total cervical lymph node dissection is not possible for each patient, it is more expected to know whether DECT can accurately predict LLNM before surgery.

Some studies [21, 22] have shown that thyroid stimulating hormone (TSH) is closely related to the occurrence and development of PTC. However, it is not clear whether there is an association between other thyroid function indicators and LLNM. For example, preoperative serum thyroglobulin (Tg), anti-thyroid stimulating hormone (Anti-Tg), anti-thyroid peroxidase (Anti-TPO), and so on.

In the current study, we hypothesized that parameters of the primary lesion from DECT and thyroid functional indicators were potentially associated with LLNM in patients with PTC. The purpose of the study was first to evaluate independent risk factors for predicting ipsilateral LLNM (ipsi-LLNM) by combining DECT and thyroid functional indicators. Second, to explore the accuracy of preoperative prediction of ipsi-LLNM combined with the above independent risk factors.

## Methods

### Patients population

**Fig. 1:**
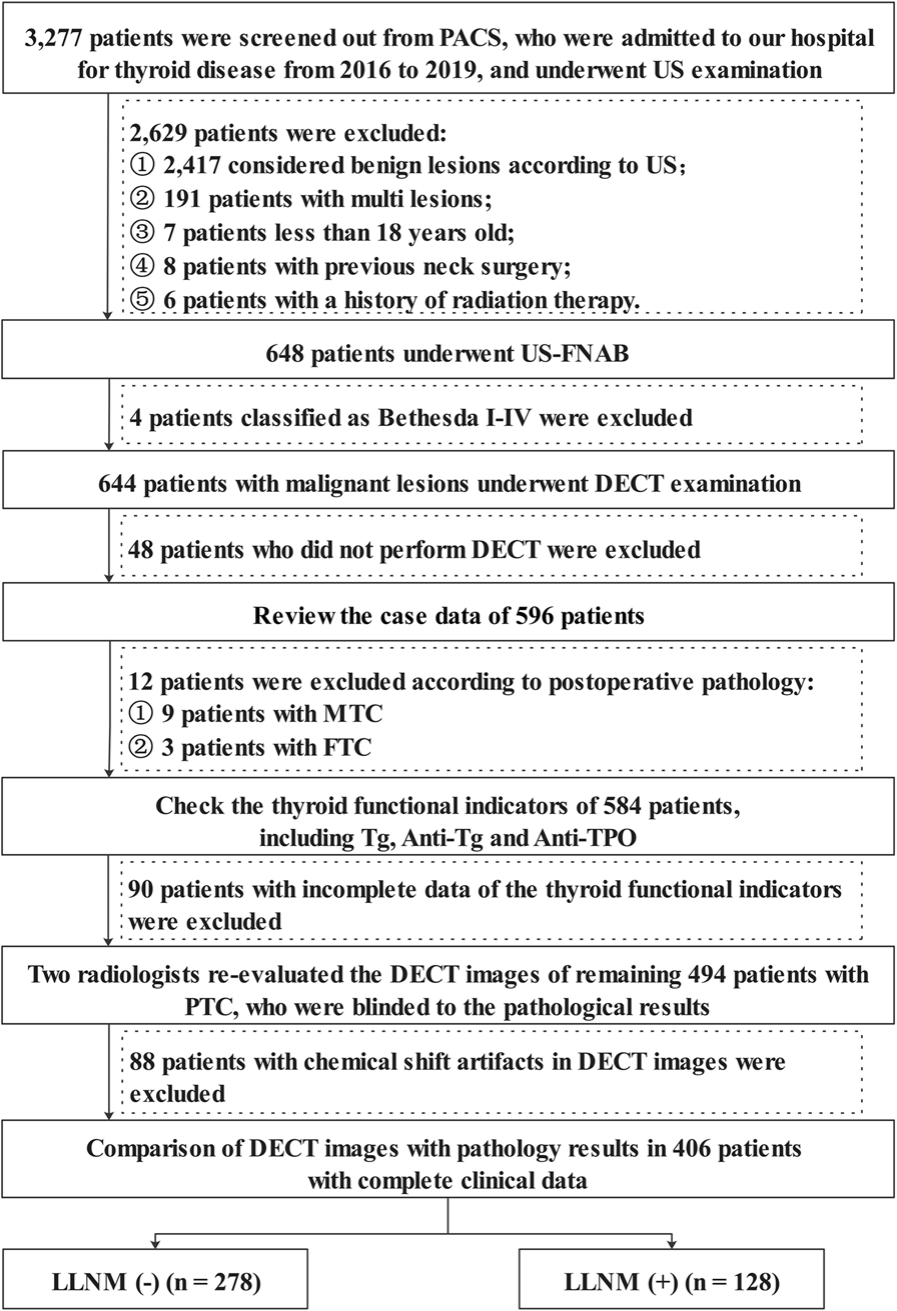
Flowchart showed criteria of inclusion and exclusion of patients with PTC in the current study. PACS = picture archiving and communication systems, US = ultrasound, FNAB = fine-needle aspiration biopsy, DECT = dual-energy computed tomography, MTC = medullary thyroid carcinoma, FTC = follicular thyroid carcinoma, PTC = papillary thyroid carcinoma, LLNM = lateral cervical lymph node metastasis

The ethics committee of Tianjin First Central Hospital approved this retrospective study (NO. 2019N153KY), and the requirement for written informed consent was waived since the retrospective nature. We reviewed the US data of 3277 patients admitted to our hospital due to thyroid disease from 2016 to 2019. According to the US diagnostic criteria of thyroid cancer in American College of Radiology (ACR) Thyroid Imaging, Reporting and Data System (TI-RADS) [23], to exclude 2629 patients considered benign lesions. The remaining 648 patients were subjected to rigorous inclusion and exclusion criteria, and 406 PTC patients (84 males, mean age, 45.86 years ±13.98; 322 females, mean age, 47.14 years ±12.56) were eventually enrolled (Fig. 1 and S1). They all underwent total thyroidectomy or thyroid lobectomy with central cervical lymph node dissection (CLND). Whether to perform LLND was determined based on preoperative imaging examination. For patients with ipsilateral LLND, we take postoperative pathology as the gold standard. For patients who did not perform LLND, we default to LLNM negative. We conducted a US follow-up for more than half a year and confirmed no suspected LLNM. For all the above 406 patients, we conducted a US follow-up for at least half a year after surgery and proved that they did not have LNM in the contralateral cervical region. Four examples were listed in Figure S2 to illustrate the criteria for the ultrasonic diagnosis of PTC and LNM. Refer to Supplement 1 for specific US and CT diagnostic criteria of cervical LNM in patients with PTC.

### Image acquisition and processing

All scans were performed using a 64 multi-detector row CT scanner (SOMATOM Definition Flash, Siemens Healthcare, Forchheim, Germany) with dual-phase contrast-enhanced CT. The detailed CT protocol was provided in Supplement 2.

### Study design

Baseline information, including age, sex, final pathology diagnosis, preoperative serum Tg, Anti-Tg, and Anti-TPO among PTC patients, was obtained from the medical record. The diagnosis of Hashimoto’s thyroiditis (HT) and nodular goiter is based on postoperative pathology as the gold standard. In this study, to describe the lesion more accurately, we used volume instead of diameter. Two radiologists with 11 and 13 years of experience in head and neck imaging diagnosis, respectively, used ImageJ software (public software, version ImageJ v1.8.0) to measure the volume of primary foci. The two radiologists also measured the DECT parameters, including cystic degeneration, calcification, extrathyroidal extension (ETE), iodine concentration (IC) in the arterial and the venous phases. They manually freehand depicted a region of interest (ROI) on three adjacent slices containing the largest lesion area to measure each lesion’s IC. ROI was placed in the substantial part of the primary lesion, covering the entire lesion as large as possible, and avoiding peripheral fat, cystic, necrosis, and calcification (Figure S3). Take the average of the three measurements as the final evaluation. Moreover, the two radiologists were blinded to clinical data and pathological results. A week later, all lesions were retested. Intra- and inter-observer consistency analyses were performed. Tumor pathology was classified according to the 2017 World Health Organization (WHO) published recommendations and the American Joint Committee on Cancer (AJCC) 8th edition [24].

### Explanation of related concepts

Different surgical procedures were chosen for thyroid lesions and lymph nodes based on preoperative imaging examination, clinical data, and the patient’s wishes. The extent of lymph node dissection was according to the Chinese Society of Clinical Oncology (CSCO) guidelines [25] and ATA guidelines [5]. Bilateral CLND included removing pre-laryngeal, pretracheal, and both the right and left paratracheal nodal basins. Ipsilateral CLND included pre-laryngeal, pretracheal, and paratracheal nodal basins on the side of the primary tumor. LLND was defined as compartment oriented functional lateral neck dissection, including levels II to V.

### Statistical analysis

Statistical analysis was performed using SPSS Statistics version 21.0 (IBM, Armonk, NY) and Medcalc 18.2.1. GraphPad prism 8.3.0 and Medcalc 18.2.1 were used to draw graphs. The chi-square analysis was calculated for categorical variables, including age, sex, location, HT, nodular goiter, cystic degeneration, calcification, and ETE. We divided the patients into two groups based on age, using 55-year-old as a cutoff value according to the 8th AJCC staging systems [26]. The t-test was used for continuous variables including Tg, Anti-Tg and Anti-TPO, volume, IC in the arterial phase, and IC in the venous phase. A consistency analysis was performed to test the agreement of quantitative parameters of DECT between the two radiologists. Statistical significance for analysis was determined to be *P* value < 0.05. Univariate analysis was performed using Student’s T-tests for normally distributed data and Mann-Whitney U test for continuous variables that were not normally distributed. We specified a priori that the variables with a *P* value < 0.05 in the univariate analysis would be candidate variables in the multivariable binary logistic regression analysis. Subsequently, candidate variables were entered as independent variables into a binary logistic backward stepwise regression analysis to select the independent predictors [27]. At each step, the variable with the highest *P* value was eliminated until the remaining variables with *P* values < 0.05. These variables acted as independent risk factors. AUC was used to evaluate the combined application of the above independent risk factors to predict the possibility of ipsi-LLNM.

## Result

### Baseline characteristics

Among 406 patients enrolled in the current study, 128 patients (31.5%) were associated with LLNM, and 112 of them (87.5%) had concurrent central cervical LNM (CLNM). Of note, there were 16 patients (12.5%) with skip metastases, meaning LLNM without CLNM. Baseline information and DECT images characteristics of primary foci according to LLNM status were summarized in Table 1. The median age was 48 years (IQR 36–57 years, range 22–77 years). The majority of patients were females (322 patients, 79.3%; 50 years, IQR 39–58 years), and 20.7% (84 patients) were males (41 years, IQR 32–55 years). One hundred forty-nine primary foci (36.7%) were with cystic degeneration, 85 primary foci (21.0%) were with calcification, and 104 primary foci (25.6%) were with ETE. The above parameters were all statistical significance for differentiation between patients with ipsi-LLNM and without (*P* < .05). Check the specific information about other parameters in Table 1. We also collected the related immunohistochemistry (IHC) and histological variant indicators of some PTC patients. See Table S1 and S2 for details.

**Table 1:** Baseline information and DECT images characteristics of PTC patients

	Total (*n* = 406)	LLNM (−) (*n* = 278)	LLNM (+) (*n* = 128)	*P* value
Age^a^				.179
≤ 55	283 (69.7)	188 (67.6)	95 (74.2)	
> 55	123 (30.3)	90 (32.4)	33 (25.8)	
Sex, n (%)				.146
Female	322 (79.3)	226 (70.2)	96 (29.8)	
Male	84 (20.7)	52 (61.9)	32 (38.1)	
Location				.493
Left lobe	189 (46.6)	127 (67.2)	62 (32.8)	
Right lobe	193 (47.5)	132 (68.4)	61 (31.6)	
Isthmus	24 (5.9)	19 (79.2)	5 (20.8)	
HT, n (%)				.270
Negative	323 (79.6)	217 (67.2)	106 (32.8)	
Positive	83 (20.4)	61 (73.5)	22 (26.5)	
Nodular Goiter, n (%)				.940
Negative	277 (68.2)	190 (68.6)	87 (31.4)	
Positive	129 (31.8)	88 (68.2)	41 (31.8)	
Cystic degeneration				.047
Negative	257 (63.3)	215 (83.7)	42 (16.3)	
Positive	149 (36.7)	63 (42.3)	86 (57.7)	
Calcification				.000
Negative	321 (79.0)	257 (80.1)	64 (19.9)	
Positive	85 (21.0)	21 (24.7)	64 (75.3)	
ETE				.000
Negative	302 (74.4)	268 (88.7)	34 (11.3)	
Positive	104 (25.6)	10 (9.6)	94 (90.4)	

*DECT* Dual-energy computed tomography, *PTC* Papillary thyroid carcinoma, *LLNM* Lateral cervical lymph node metastasis, *HT* Hashimoto’s thyroiditis, *ETE* Extrathyroidal extension

^a^ We divided the patients into two groups based on age using 55-year-old as a cutoff value according to the 8th AJCC staging systems

### Result of consistency analysis

The intraclass correlation coefficient (ICC) calculated for the agreement of features extracted by two radiologists ranged from 0.913 to 0.974, reflecting good agreement (*P* .000). The inter- and intra-observer consistency analysis for all the parameters was more significant than 0.8, which showed the right consistency (Table S3).

### Comparison of DECT imaging parameters and thyroid function indicators between patients with and without ipsi-LLNM

**Fig. 2:**
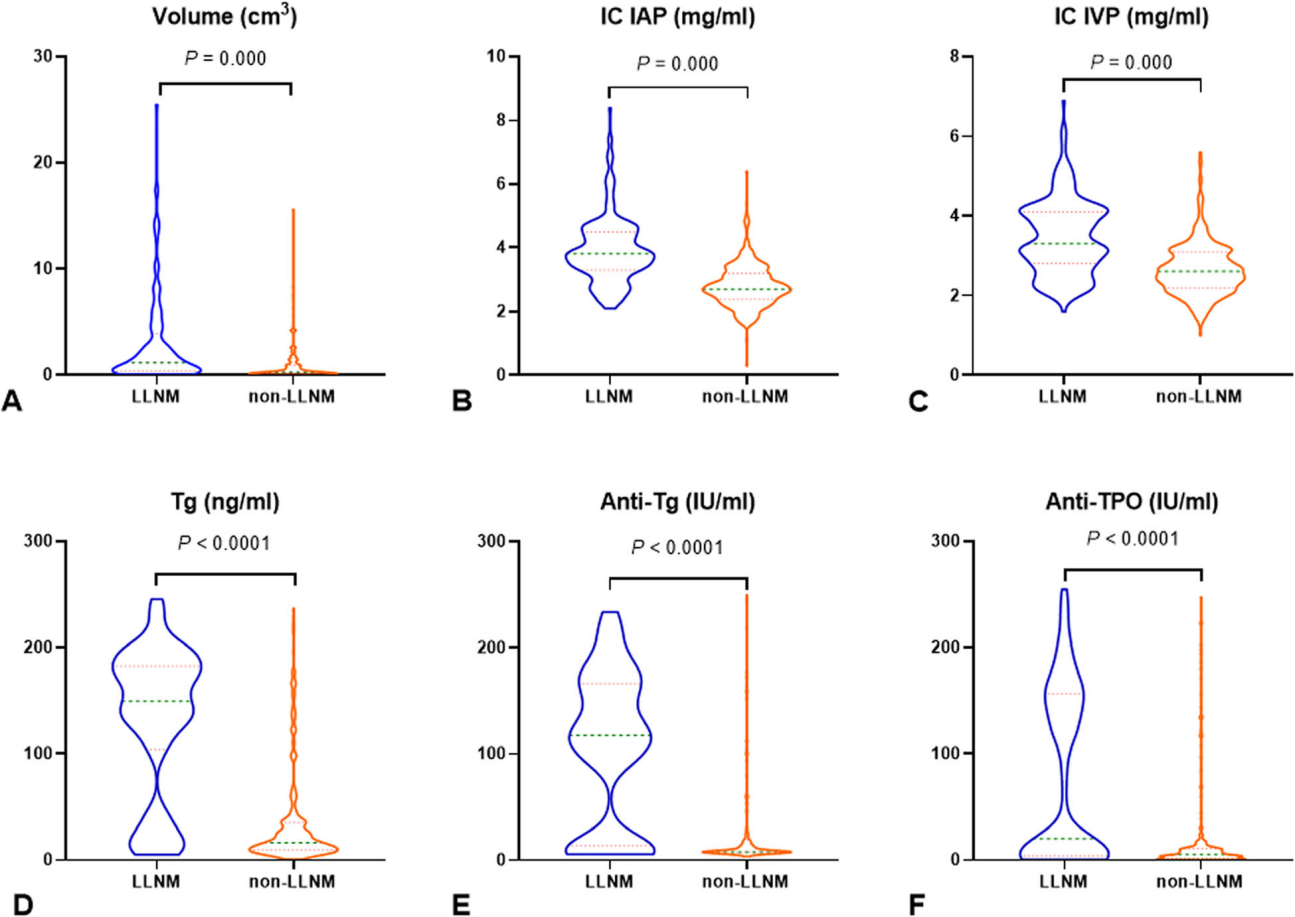
Violin plots showed that volume (**a**), IC in the arterial phase (**b**), IC in the venous phase (**c**), Tg (**d**), Anti-Tg (**e**), and Anti-TPO (**f**) were statistically significant in predicting non-LLNM and LLNM. LLNM = lateral cervical lymph node metastasis, IC = iodine concentration, IAP = in the arterial phase, IVP = in the venous phase

Quantitative parameters of patients with and without ipsi-LLNM were listed in Table 2. Tg, Anti-Tg, Anti-TPO, volume, IC in the arterial phase, and IC in the venous phase were higher in those with ipsi-LLNM than those without (*P* < .0001) (Table 2 and Fig. 2).

**Table 2:** DECT parameters and thyroid function indicators in PTC patients

	Total (*n* = 406)	LLNM (−) (*n* = 278)	LLNM (+) (*n* = 128)	*P* value
Tg (ng/mL)	25.54 (12.08–134.12)	16.28 (9.55–35.46)	149.26 (104.05–182.76)	< .0001
Anti-Tg (IU/mL)	11.5 (7.70–110.02)	7.70 (7.70–19.21)	117.70 (13.50–166.40)	< .0001
Anti-TPO (IU/mL)	7.01 (3.31–18.12)	5.24 (1.52–10.70)	19.94 (3.70–156.56)	< .0001
Volume (cm^3^)	0.38 (0.14–1.44)	0.18 (0.01–0.70)	0.72 (0.18–2.76)	.000
IC IAP (mg/mL)	3.0 (2.6–3.7)	2.8 (2.4–3.2)	3.8 (3.3–4.5)	.000
IC IVP (mg/mL)	2.8 (2.4–3.3)	2.6 (2.2–3.1)	3.3 (2.8–4.1)	.000

Note: Mann-Whitney U test, median (IQR)

*DECT* Dual-energy computed tomography, *PTC* Papillary thyroid carcinoma, *LLNM* Lateral cervical lymph node metastasis, *IC* Iodine concentration, *IAP* In the arterial phase, *IVP* In the venous phase, *IQR* Interquartile range

### Univariate and multivariate logistic regression analysis of risk factors for ipsi-LLNM in patients with PTC

The univariable analysis showed that Tg, Anti-Tg, volume, cystic degeneration, calcification, IC in the arterial phase, IC in the venous phase, and ETE were risk factors for predicting the possibility of ipsi-LLNM (*P* range, .000–.006). Further multivariable logistic regression analysis showed that among these parameters, Tg (OR, 2.668; 95% CI: 1.590, 4.475; *P* .000), Anti-Tg (OR, 2.001; 95% CI: 1.202, 3.333; *P* .008), ETE (OR, 6.335; 95% CI: 3.768, 10.651; *P* .000), IC in the arterial phase (OR, 3.691; 95% CI: 2.170, 6.278; *P* .000), and IC in the venous phase (OR, 2.122; 95% CI: 1.271, 3.541; *P* .004) were the independent predictors for ipsi-LLNM. Sex, age, Anti-TPO, HT, nodular goiter, volume, cystic degeneration, and calcification were not related to ipsi-LLNM in patients with PTC (*P* > .05) (Table 3).

**Table 3:** Risk factors for ipsi-LLNM in patients with PTC

	Univariate analysis			Multivariate analysis		
	OR	95% CI	*P* value	OR	95% CI	*P* value
Sex	0.690	0.418–1.139	.147			
Age	0.726	0.454–1.160	.180			
Tg (ng/mL)	2.832	1.837–4.365	.000	2.668	1.590–4.475	.000
Anti-Tg (IU/mL)	2.121	1.386–3.245	.001	2.001	1.202–3.333	.008
Anti-TPO (IU/mL)	1.054	0.693–1.603	.805			
HT	0.738	0.430–1.267	.271			
Nodular goiter	1.018	0.649–1.594	.940			
Volume (cm^3^)	1.093	1.029–1.161	.004			
Cystic degeneration	1.816	1.190–2.771	.006			
Calcification	1.989	1.298–3.048	.002			
IC IAP (mg/mL)	4.418	2.787–7.004	.000	3.691	2.170–6.278	.000
IC IVP (mg/mL)	2.517	1.640–3.863	.000	2.122	1.271–3.541	.004
ETE	6.350	4.012–10.050	.000	6.335	3.768–10.651	.000

*LLNM* Lateral lymph node metastasis, *PTC* Papillary thyroid carcinoma, *OR* Odds ratio, *CI* Confidence interval, *HT* Hashimoto’s thyroiditis, *ETE* Extrathyroidal extension, *IC* Iodine concentration, *IAP* In the arterial phase, *IVP* In the venous phase

### The cutoff value of each parameter for predicting ipsi-LLNM in PTC patients

The AUC, sensitivity, specificity, positive predictive value (PPV), and negative predictive value (NPV) for differentiating ipsi-LLNM for each parameter were listed in Table 4. ROC curve analysis determined that the optimal cutoff points for Tg, Anti-Tg, IC in the arterial phase, and IC in the venous phase in predicting ipsi-LLNM were 100.01 ng/mL (AUC 0.856, 95%CI 0.818–0.889), 89.43 IU/mL (AUC 0.766, 95%CI 0.721–0.806), 3.4 mg/mL (AUC 0.846, 95%CI 0.807–0.879), and 3.1 mg/mL (AUC 0.777, 95%CI 0.733–0.816), respectively (Figure S4). The specific information on other indicators was listed in Table 4.

**Table 4:** The performance of each risk factor to predict ipsi-LLNM in patients with PTC

Parameters	Cutoff value	AUC (95%CI)	Sensitivity (%)	Specificity (%)	PPV (%)	NPV (%)	*P* Value
Tg (ng/mL)	> 100.01	0.856 (0.818, 0.889)	76.56 (68.3, 83.6)	88.85 (84.5, 92.3)	76.0 (69.1, 81.7)	89.2 (85.7, 91.9)	< .0001
Anti-Tg (IU/mL)	> 89.43	0.766 (0.721, 0.806)	71.09 (62.4, 78.8)	86.33 (81.7, 90.1)	70.5 (63.6, 76.7)	86.6 (83.1, 89.5)	< .0001
IC IAP (mg/mL)	> 3.4	0.846 (0.807, 0.879)	72.66 (64.1, 80.2)	85.97 (81.3, 89.8)	70.5 (63.6. 76.5)	87.2 (83.7, 90.1)	< .0001
IC IVP (mg/mL)	> 3.1	0.777 (0.733, 0.816)	53.91 (44.9, 62.8)	91.37 (87.4, 94.4)	74.2 (65.5, 81.3)	81.2 (78.1, 83.9)	< .0001
ETE	N/A	0.713 (0.667, 0.757)	66.41 (57.5, 74.5)	76.26 (70.8, 81.1)	56.3 (50.2, 62.2)	83.1 (79.3, 86.4)	< .0001

^*^ Data in parentheses are 95% confidence intervals (CIs)

*LLNM* Lateral cervical lymph node metastasis, *PTC* Papillary thyroid carcinoma, *AUC* Area under the curve, *PPV* Positive predictive value, *NPV* Negative predictive value, *IC* Iodine concentration, *IAP* In the arterial phase, *IVP* In the venous phase, *ETE* Extrathyroidal extension

### Combined DECT and thyroid functional indicators to predict the performance of ipsi-LLNM

**Fig. 3:**
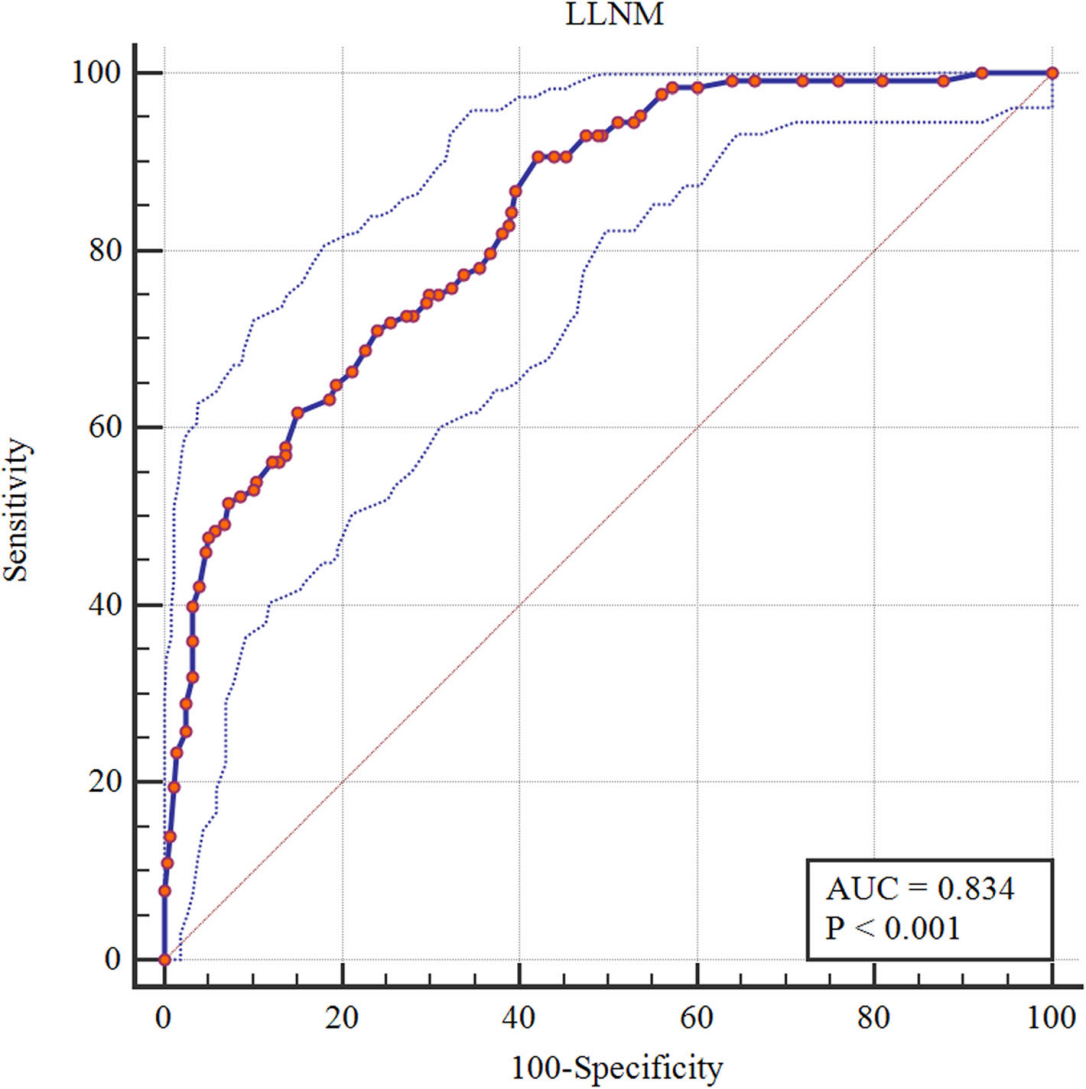
ROC curve of combined DECT parameters and thyroid function indicators in patients with PTC. ROC = receiver operating characteristic, DECT = dual-energy computed tomography, PTC = papillary thyroid carcinoma

**Fig. 4:**
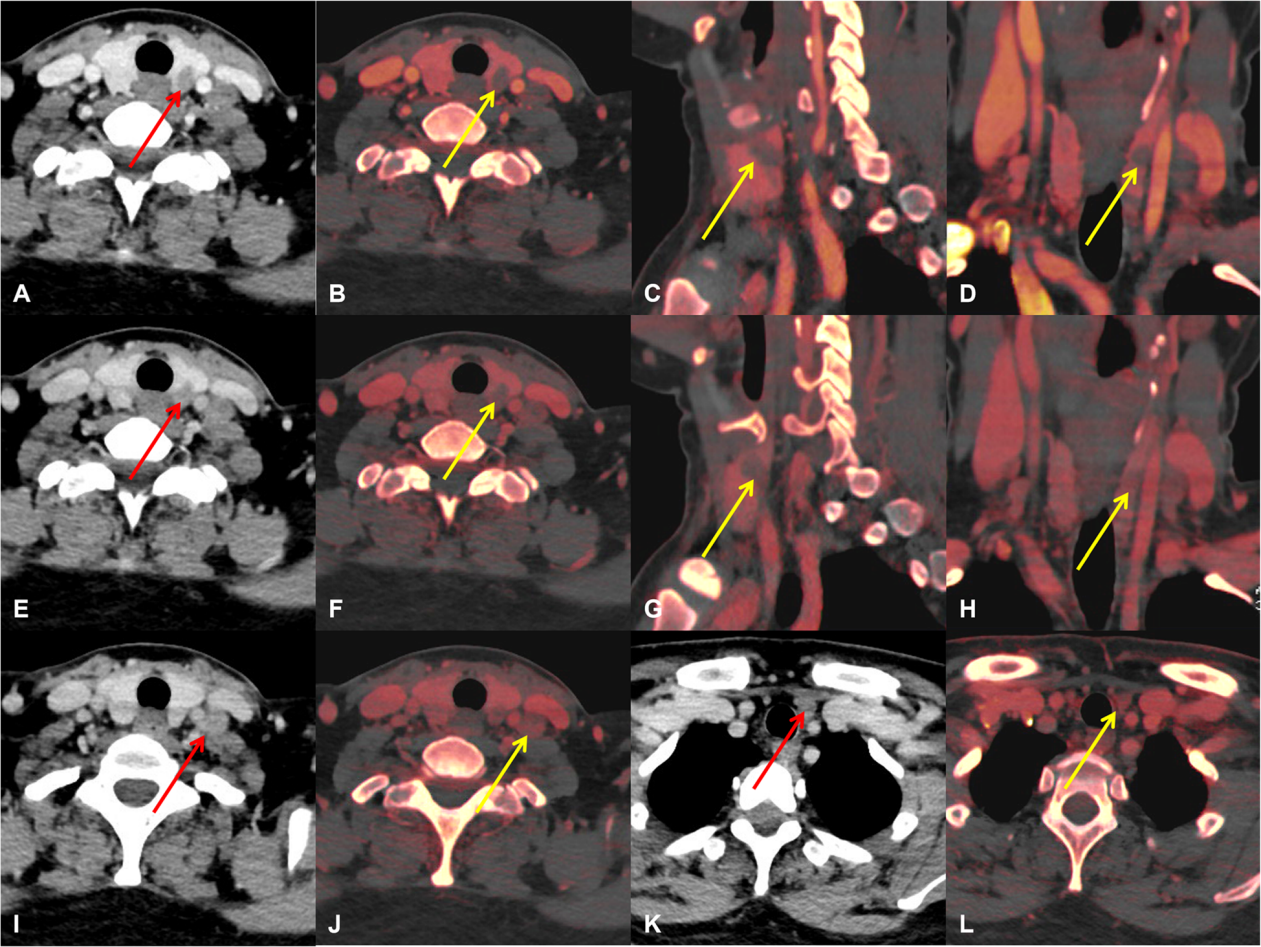
An example of a PTC patient with LLNM. Total thyroidectomy with left lateral level II-VI dissection was performed. Postoperative pathology confirmed PTC with a diameter of 1.1 cm, and five and four metastatic lymph nodes were found in the left level IV and VI, respectively. **a**, **e**, A primary lesion located in the left lobe of thyroid on the contrast-enhanced arterial phase (**a**) and venous phase (**e**). **b**-**d**, The iodine maps of the primary lesion in the axial, sagittal, and coronal positions in the arterial phase (IC = 3.5 mg/mL). **f**-**h**, The iodine maps of the primary lesion in the axial, sagittal, and coronal positions in the venous phase (IC = 2.9 mg/mL). **i**, **j**, An enlarged lymph node located in the left level IVa in the arterial phase image and iodine map. **k**, **l**, Another enlarged lymph node located in the left level VIb in the arterial phase image and iodine map. PTC = papillary thyroid carcinoma, LLNM = lateral cervical lymph node metastasis, IC = iodine concentration

**Fig. 5:**
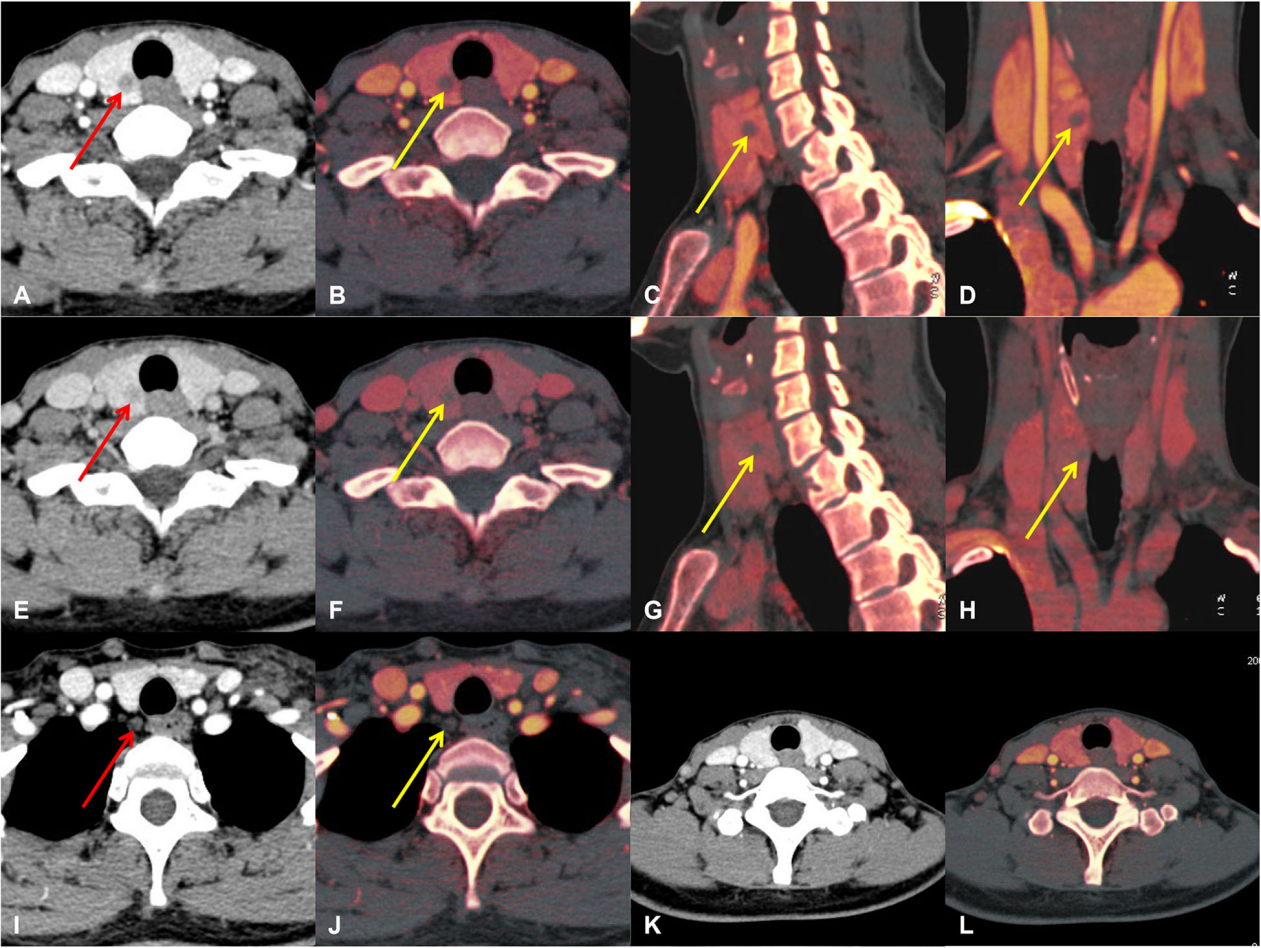
An example of a PTMC patient without LLNM. The right lobe and isthmus of the thyroid were excised, and lymph nodes in the right level VI were dissected. Postoperative pathology confirmed PTMC accompanied by HT, with a diameter of 0.9 cm, and no metastatic lymph nodes were found in the right level VI. **a**, **e**, A primary lesion located in the right lobe of thyroid on the contrast-enhanced arterial phase (**a**) and venous phase (**e**). **b**-**d**, The iodine maps of the primary lesion in the axial, sagittal, and coronal positions in the arterial phase (IC = 1.1 mg/mL). **f**-**h**, The iodine maps of the primary lesion in the axial, sagittal, and coronal positions in the venous phase (IC = 1.0 mg/mL). **i**, **j**, A benign lymph node located in the right level VIb on the arterial phase and iodine map. **k**, **l**, No metastatic lymph nodes were found in the lateral cervical region on the CT images. PTMC = papillary thyroid microcarcinoma, LLNM = lateral cervical lymph node metastasis, HT = Hashimoto’s thyroiditis, IC = iodine concentration, CT = computed tomography

The AUC of combined DECT quantitative parameters and thyroid function indicators in patients with PTC was 0.834 (95%CI 0.795–0.869), indicating that the combined application of the independent risk factors helped predict the ipsi-LLNM (Fig. 3). Furthermore, there were three examples of predicting ipsi-LLNM, which might help illustrate these independent risk factors (Figs. 4, 5, and Figure S5). We further gave another two examples. In both cases, the general CT features mispredicted the possibility of LLNM, but when the quantitative parameters of DECT and thyroid function indicators were combined, the chance of LLNM could be accurately predicted, thus further explaining the significance of this study (Figure S6 and S7).

## Discussion

In this retrospective study, we analyzed the effects of DECT quantitative parameters and thyroid function indicators on ipsi-LLNM in patients with PTC to determine the independent risk factors for predicting the possibility of ipsi-LLNM. There were three significant findings. First, the IC of the primary lesions in the arterial phase > 3.4 mg/mL and IC in the venous phase > 3.1 mg/mL were positively associated with the risk of ipsi-LLNM in PTC patients. Second, Tg >  100.01 ng/mL and Anti-Tg >  89.43 IU/mL were two independent risk factors for ipsi-LLNM. Third, the combined application of DECT quantitative parameters and thyroid functional indicators might improve the diagnostic performance in the prediction of ipsi-LLNM, with an AUC of 0.843.

In the current study, IC of the primary lesion in the arterial and the venous phases were independent risk factors for ipsi-LLNM in patients with PTC. In Liu’s study [17], DECT was used to assess LNM in PTC patients quantitatively. Compared with their research, we had a much larger sample size (406 vs. 52), and the AUC of IC in the arterial phase in our study (0.846) was slightly higher than theirs (0.811). Moreover, we chose the primary focus as the prediction target, reducing the possible errors caused by pathology and lymph node one-to-one correspondence. As well known, IC is a highly sensitive and specific parameter for identifying benign and malignant thyroid nodes [28, 29], which is a direct response to blood flow and is affected by the number of blood vessels [30]. Normal follicular cells are responsible for thyroid iodine uptake in benign conditions, whereas, in PTC, they are replaced by cancer cells or fibrous tissues. The specific iodine absorption characteristics of thyroid tissue and the changed in tumor-related vascular patterns in lymph nodes are also correlated with IC. Therefore, the differences in the iodine uptake might lead to differences in the lymph nodes metastatic capacity. We speculated that the higher the IC of the primary foci, the greater the probability of ipsi-LLNM.

Tg was a vital tumor marker for PTC patients [31]. Furthermore, there was a mutual influence between Tg and Anti-Tg [32]. Anti-Tg and Anti-TPO were closely related to the occurrence of PTC [33]. Most previous studies had demonstrated that PTC might indeed lead to an autoimmune reaction characterized by circulating thyroid function indicators [34]. However, whether these indicators could be potential predictive factors of ipsi-LLNM has not been proved. In the current study, univariate analysis results suggested that Tg and Anti-Tg were related to ipsi-LLNM (*P* range, .000–.001). The further multivariate logistic analysis showed that Tg > 100.01 ng/mL and Anti-Tg > 89.43 IU/mL were both independent risk factors for ipsi-LLNM in agreement with Li’s reports [21]. Based on these results, we conclude that Tg and Anti-Tg might be correlated with tumor aggressiveness and prognosis in patients with PTC. The measurement could give additional information for predicting aggressiveness and ipsi-LLNM. Therefore, we suggest that surgeons should pay more attention to the levels of Tg and Anti-Tg, which may have potential predictive value for ipsi-LLNM.

Besides, ETE was another independent predictor for ipsi-LLNM, which was consistent with previous studies [35–40]. We considered that the more aggressive the tumor, the greater the probability of LNM.

In the current study, of 128 patients with LLNM, 16 patients developed skip metastasis (12.5%), which was consistent with previous research [41–44]. Unfortunately, due to the small number of cases, in this study, we cannot count the risk factors related to skip metastasis. In the future, after expanding the sample size, we will do further research.

The present study has some limitations due to its retrospective design. First, because of its retrospective nature, the inspection items could not be designed beforehand. Therefore, we cannot get accurate postoperative pathological information about the size of metastatic lymph nodes, so we cannot predict micro- or macro-metastasis. In the future, we will conduct prospective studies to solve this problem. Second, for patients without preoperative imaging evidence to support the presence of LLNM, LLND was not performed after obtaining the informed consent of the patients. These patients were classified as LLNM (−) by default, leading to potential selection bias. However, we have conducted a US follow-up of at least half a year for these patients, and it was confirmed that there is no LLNM. Therefore, we also ensure the reliability and accuracy of this study to a certain extent. Third, also due to the retrospective nature, IHC indicators and histological variants have not been collected completely, which might have a positive effect on the prediction of LLNM in PTC patients. Fourth, to avoid the multiple lesions’ mutual influence, this study only included PTC patients with a single lesion. In the future, we will consist of patients with multiple bilateral lesions for more in-depth research. To sum up, a multicenter, large sample, and prospective clinical trials should be performed to identify the predicting factors of LLNM in patients with PTC and provide more supporting evidence with more excellent reliability.

## Conclusion

We demonstrated that combining the DECT quantitative parameters and thyroid function indicators could effectively predict ipsi-LLNM in PTC patients. This strategy may be an effective assist for clinicians to formulate surgical procedures before surgery accurately. With further verification in a larger population and prospective study, our result has great potential to serve as an essential decision support tool in clinical applications.

## Supplementary Information

**Additional file 1: Supplement 1.** Specific ultrasound and dual-energy computed tomography (DECT) diagnostic criteria of cervical lymph node metastasis in patients with papillary thyroid carcinoma (PTC). **Supplement 2.** DECT Examination. **Table S1.** IHC indicators and the histological variants of some PTC patients. **Table S2.** The histological variation information of some PTC patients included in the current study. **Table S3.** The consistency analysis of the measurement indexes of the two readers. **Figure S1.** Flowchart of the diagnosis of LLNM. **Figure S2.** Ultrasonic diagnostic standards for PTC and LNM. **Figure S3.** The ROI of the primary lesion on the DECT images. **Figure S4.** ROC curves of DECT parameters and thyroid function indicators in patients with PTC. **Figure S5.** Example 1 of using DECT to predict LLNM. **Figure S6.** Example 2 of using DECT to predict LLNM. **Figure S7.** Example 3 of using DECT to predict LLNM.

## Data Availability

The data that support the findings of this study are available from Tianjin First Central Hospital, but restrictions apply to the availability of these data, which were used under license for the current research, and so are not publicly available. Data are, however, available from the corresponding authors upon reasonable request and with permission of Tianjin First Central Hospital.

## Abbreviations

PTC: Papillary thyroid carcinoma
LNM: Lymph node metastasis
ATA: American Thyroid Association
LLND: Lateral cervical lymph node dissection
LLNM: Lateral cervical lymph node metastasis
US: Ultrasound
DECT: Dual-energy computed tomography
NCCN: National Comprehensive Cancer Network
RAI: Radioactive iodine
λ_HU_: The slope of the energy spectrum curve
NIC: Normalized iodine concentration
ROC: Receiver operating characteristic
AUC: Area under the curve
TSH: Thyroid stimulating hormone
Tg: Thyroglobulin
Anti-Tg: anti-thyroid stimulating hormone
Anti-TPO: Anti-thyroid peroxidase
ipsi-LLNM: Ipsilateral lateral cervical lymph node metastasis
ACR: American College of Radiology
TI-RADS: Thyroid Imaging, Reporting and Data System
CLND: Central cervical lymph node dissection
HT: Hashimoto’s thyroiditis
ETE: Extrathyroidal extension
IC: Iodine concentration
ROI: Region of interest
WHO: World Health Organization
AJCC: American Joint Committee on Cancer
CSCO: Chinese Society of Clinical Oncology
CLNM: Central cervical lymph node metastasis
IQR: Interquartile range
IHC: Immunohistochemistry
ICC: Intraclass correlation coefficient
PPV: Positive predictive value
NPV: Negative predictive value
CI: Confidence interval
