# Supplementary material for: Prediction of ipsilateral lateral cervical lymph node metastasis in papillary thyroid carcinoma: a combined dual-energy CT and thyroid function indicators study
Source: BMC Cancer. 2021 Mar 4;21:221. doi: 10.1186/s12885-021-07951-0 (PMC7934388; doi:10.1186/s12885-021-07951-0)
Supplement: Supplementary file 1 — Additional file 1: Supplement 1. Specific ultrasound and dual-energy computed tomography (DECT) diagnostic criteria of cervical lymph node metastasis in patients with papillary thyroid carcinoma (PTC). Supplement 2. DECT Examination. Table S1. IHC indicators and the histological variants of some PTC patients. Table S2. The histological variation information of some PTC patients included in the current study. Table S3. The consistency analysis of the measurement indexes of the two readers. Figure S1. Flowchart of the diagnosis of LLNM. Figure S2. Ultrasonic diagnostic standards for PTC and LNM. Figure S3. The ROI of the primary lesion on the DECT images. Figure S4. ROC curves of DECT parameters and thyroid function indicators in patients with PTC. Figure S5. Example 1 of using DECT to predict LLNM. Figure S6. Example 2 of using DECT to predict LLNM. Figure S7. Example 3 of using DECT to predict LLNM. [file 12885_2021_7951_MOESM1_ESM.docx]

**Supplement**

**Supplement 1: Specific ultrasound and dual-energy computed tomography (DECT) diagnostic criteria of cervical lymph node metastasis in patients with papillary thyroid carcinoma (PTC)**

Sonographic features suggestive of abnormal metastatic lymph nodes, according to 2015 American Thyroid Association (ATA) Management Guidelines for Adult Patients with Thyroid Nodules and Differentiated Thyroid Cancer (1), includes enlargement, loss of the fatty hilum, a rounded rather than oval shape, hyperechogenicity, cystic change, calcification, and peripheral vascularity. The location of the lymph nodes may also be useful for decision-making. Malignant lymph nodes are much more likely to occur in levels III, IV, and VI than in level II. No single sonographic feature is adequately sensitive for detecting lymph nodes with metastatic thyroid cancer (2).

Metastatic lymph nodes were deemed to be present in CT images when at least one of the following criteria was fulfilled: size larger than or equal to 10 mm, substantial enhancement, heterogeneous enhancement, calcification, cystic or necrotic change, and extrathyroidal extension (ETE) (3, 4). Lymph node size was determined by using the maximal short-axis diameter. Strong enhancement was considered to be similar to that of the pharyngeal mucosa. Fuzzy boundaries or invasion into adjacent tissues were considered to indicate ETE (5).

**Supplement 2: DECT Examination**

Patients held their breath during eupnea before the horizontal scan in a transverse position. All patients were scanned craniocaudally in the supine position with the bilateral upper limbs placed on both sides, shoulders drooping as much as possible, head slightly tilted. Moreover, the longitudinal alignment of the positioning cursor was aligned on the central sagittal plane of the cervicothoracic region. The orthotopic scanning was performed firstly, and then the scanning baseline and range were confirmed according to the scout view. The whole neck was scanned from the upper edge of the aortic arch to the lower edge of the submandibular gland, which covered the thyroid and cervical lymph nodes area.

Keep the patient’s heart rate at an average level throughout the scan. After CT scanning, arterial phase and venous phase contrast-enhanced scanning were performed. The images were acquired in the dual-energy mode by using the following parameters: tube current 600 mA, helical thickness 6 mm, helical pitch 0.9, rotation speed 0.28 s, detector width 40 mm, collimation 64×0.6 mm. The scan parameters were set according to the concept of as low as reasonably achievable for radiation protection. A fast rotation speed and a moderate helical pitch were chosen to obtain fast scanning speed and to reduce motion artifacts of neck and radiation dose. For contrast-enhanced scanning, an iodinated nonionic contrast agent (iohexol; 350 mg/dl iodine, SOMATOM Definition Flash, Siemens Healthcare, Forchheim, Germany) was administered through the right elbow median vein by a dual-head injector. The dosage was 1 ml/kg with a flow rate of 3 ml/s. The total injection dose was 60 - 70 ml, followed by a bolus injection of 40 ml saline given at the same flow rate. The arterial phase scanning was determined by automatic trigger technique, and the scanning delay was 25 s at the beginning of arterial phase scanning. The delay time of the venous scan was 20 s after the end of the arterial scan.

All the original CT data were reconstructed into contiguous axial images with a section thickness of 1 mm, a field of view (FOV) of 200 mm, and a matrix of 512 × 512. The DECT data of arterial and venous phases were transferred to the SIEMENS Syngovia workstation (Syngo DE, Siemens Healthcare, Forchheim, Germany) for analysis, the Liver VNC function keys for computer automatic processing, and then the iodine maps were obtained.

**Table S1: IHC indicators and the histological variants of some PTC patients**

|  | No. of PTC patients involved | LLNM | |  | CLNM | |
| --- | --- | --- | --- | --- | --- | --- |
|  |  | LLNM (-) | LLNM (+) |  | CLNM (-) | CLNM (+) |
| **IHC indicators** |  |  |  |  |  |  |
| HBME-1 (-) | 91/406 (22.4) | 73 (80.2) | 18 (19.8) |  | 69 (75.8) | 22 (24.2) |
| HBME-1 (+) | 36/406 (8.9) | 11 (30.6) | 25 (69.4) |  | 5 (13.9) | 31 (86.1) |
| ALK (-) | 31/406 (7.6) | 26 (83.9) | 5 (16.1) |  | 24 (77.4) | 7 (22.6) |
| ALK (+) | 12/406 (3.0) | 4 (33.3) | 8 (66.7) |  | 3 (25.0) | 9 (75.0) |
| Galectin-3 (-) | 79/406 (19.5) | 62 (78.5) | 17 (21.5) |  | 59 (74.7) | 20 (25.3) |
| Galectin-3 (+) | 23/406 (5.7) | 7 (30.4) | 16 (69.6) |  | 3 (13.0) | 20 (87.0) |
| CK 19 (-) | 55/406 (13.5) | 41 (74.5) | 14 (25.5) |  | 34 (61.8) | 21 (38.2) |
| CK 19 (+) | 21/406 (5.2) | 13 (61.9) | 8 (38.1) |  | 8 (38.1) | 13 (61.9) |
| CD 56 (-) | 52/406 (12.8) | 39 (75.0) | 13 (25.0) |  | 35 (67.3) | 17 (32.7) |
| CD 56 (+) | 29/406 (7.1) | 18 (62.1) | 11 (37.9) |  | 16 (55.2) | 13 (44.8) |
| **Histological variants** | |  |  |  |  |  |
| Classical variant | 6/406 (1.5) | 4 (66.7) | 2 (33.3) |  | 2 (33.3) | 4 (66.7) |
| Follicular variant | 17/406 (4.2) | 13 (76.5) | 4 (23.5) |  | 7 (41.2) | 10 (58.8) |

Note: Percentage in brackets

IHC = immunohistochemistry, PTC = papillary thyroid carcinoma, LLNM = lateral cervical lymph node metastasis, CLNM = central cervical lymph node metastasis, HBME-1 = Hector Battifora mesothelial-1, ALK = anaplastic lymphoma kinase, CK 19 = Cytokeratin 19, CD 56 = Cluster of Differentiation 56

**Table S2: The histological variation information of some PTC patients included in the current study**

| Histological variants | NO | PTC and LNM |
| --- | --- | --- |
| Classical variant | 1 | PTC in the right lobe, the diameter of 1 cm, without LNM |
|  | 2 | PTC in the left lobe, the diameter of 1.2 cm, left Ⅲ (1/6) |
|  | 3 | PTC in the left lobe, the diameter of 1.4 cm, left Ⅵ (1/3) |
|  | 4 | PTC in the right lobe, the diameter of 0.6 cm, right Ⅵ (6/7) |
|  | 5 | PTC in right lobe, diameter of 1 cm, right Ⅵ (2/3)，right Ⅲ/Ⅳ (1/10) |
|  | 6 | PTC in the right lobe, the diameter of 0.9 cm，right Ⅵ (4/9) |
| Follicular variant | 1 | PTC in the right lobe, the diameter of 0.8 cm, right Ⅵ (2/3) |
|  | 2 | PTC in the right lobe, the diameter of 0.7 cm, without LNM |
|  | 3 | PTC in the right lobe, the diameter of 1.4 cm, right Ⅵ (2/3) |
|  | 4 | PTC in the left lobe, the diameter of 1.7 cm, right Ⅵ (2/14), right Ⅲ (3/13), left Ⅵ (2/6), right Ⅳ/Ⅴ (2/6) |
|  | 5 | PTC in the left lobe, the diameter of 1.1 cm, without LNM |
|  | 6 | PTC in the left lobe, the diameter of 0.4 cm, left Ⅵ (2/4) |
|  | 7 | PTC in the right lobe, the diameter of 1.3 cm, right Ⅵ (1/3), right Ⅱ (1/9) |
|  | 8 | PTC in the left lobe, the diameter of 1.4 cm, without LNM |
|  | 9 | PTC in the right lobe, the diameter of 1 cm, without LNM |
|  | 10 | PTC in the right lobe, the diameter of 0.8 cm, without LNM |
|  | 11 | PTC in the right lobe, the diameter of 0.8 cm, without LNM |
|  | 12 | PTC in the right lobe, the diameter of 0.2cm, left Ⅲ (1/6) |
|  | 13 | PTC in the left lobe, the diameter of 1.2 cm, left Ⅵ (1/11), right Ⅵ (4/10) |
|  | 14 | PTC in the right lobe, the diameter of 1.5 cm, right Ⅲ (1/3), right Ⅳ (2/9) |
|  | 15 | PTC in the left lobe, the diameter of 0.8 cm, without LNM |
|  | 16 | PTC in the right lobe, the diameter of 0.9 cm, right Ⅵ (1/3) |
|  | 17 | PTC in the right lobe, the diameter of 0.6 cm, without LNM |

PTC = papillary thyroid carcinoma, LNM = lymph node metastasis

**Table S1: IHC indicators and the histological variants of some PTC patients**

|  | No. of PTC patients involved | LLNM | |  | CLNM | |
| --- | --- | --- | --- | --- | --- | --- |
|  |  | LLNM (-) | LLNM (+) |  | CLNM (-) | CLNM (+) |
| **IHC indicators** |  |  |  |  |  |  |
| HBME-1 (-) | 91/406 (22.4) | 73 (80.2) | 18 (19.8) |  | 69 (75.8) | 22 (24.2) |
| HBME-1 (+) | 36/406 (8.9) | 11 (30.6) | 25 (69.4) |  | 5 (13.9) | 31 (86.1) |
| ALK (-) | 31/406 (7.6) | 26 (83.9) | 5 (16.1) |  | 24 (77.4) | 7 (22.6) |
| ALK (+) | 12/406 (3.0) | 4 (33.3) | 8 (66.7) |  | 3 (25.0) | 9 (75.0) |
| Galectin-3 (-) | 79/406 (19.5) | 62 (78.5) | 17 (21.5) |  | 59 (74.7) | 20 (25.3) |
| Galectin-3 (+) | 23/406 (5.7) | 7 (30.4) | 16 (69.6) |  | 3 (13.0) | 20 (87.0) |
| CK 19 (-) | 55/406 (13.5) | 41 (74.5) | 14 (25.5) |  | 34 (61.8) | 21 (38.2) |
| CK 19 (+) | 21/406 (5.2) | 13 (61.9) | 8 (38.1) |  | 8 (38.1) | 13 (61.9) |
| CD 56 (-) | 52/406 (12.8) | 39 (75.0) | 13 (25.0) |  | 35 (67.3) | 17 (32.7) |
| CD 56 (+) | 29/406 (7.1) | 18 (62.1) | 11 (37.9) |  | 16 (55.2) | 13 (44.8) |
| **Histological variants** | |  |  |  |  |  |
| Classical variant | 6/406 (1.5) | 4 (66.7) | 2 (33.3) |  | 2 (33.3) | 4 (66.7) |
| Follicular variant | 17/406 (4.2) | 13 (76.5) | 4 (23.5) |  | 7 (41.2) | 10 (58.8) |

Note: Percentage in brackets

IHC = immunohistochemistry, PTC = papillary thyroid carcinoma, LLNM = lateral cervical lymph node metastasis, CLNM = central cervical lymph node metastasis, HBME-1 = Hector Battifora mesothelial-1, ALK = anaplastic lymphoma kinase, CK 19 = Cytokeratin 19, CD 56 = Cluster of Differentiation 56

**Table S3: The consistency analysis of the measurement indexes of the two readers**

| Parameters | ICC (95%CI) | *P* Value |
| --- | --- | --- |
| IC in the arterial phase |  |  |
| Doctor A | 0.974(0.970-0.978) | 0.000 |
| Doctor B | 0.964(0.958-0.969) | 0.000 |
| Doctor A and doctor B | 0.916(0.902-0.928) | 0.000 |
| IC in the venous phase |  |  |
| Doctor A | 0.963(0.957-0.969) | 0.000 |
| Doctor B | 0.923(0.910-0.934) | 0.000 |
| Doctor A and doctor B | 0.913(0.899-0.926) | 0.000 |

ICC = intraclass correlation coefficient, CI = confidence interval, IC = iodine concentration


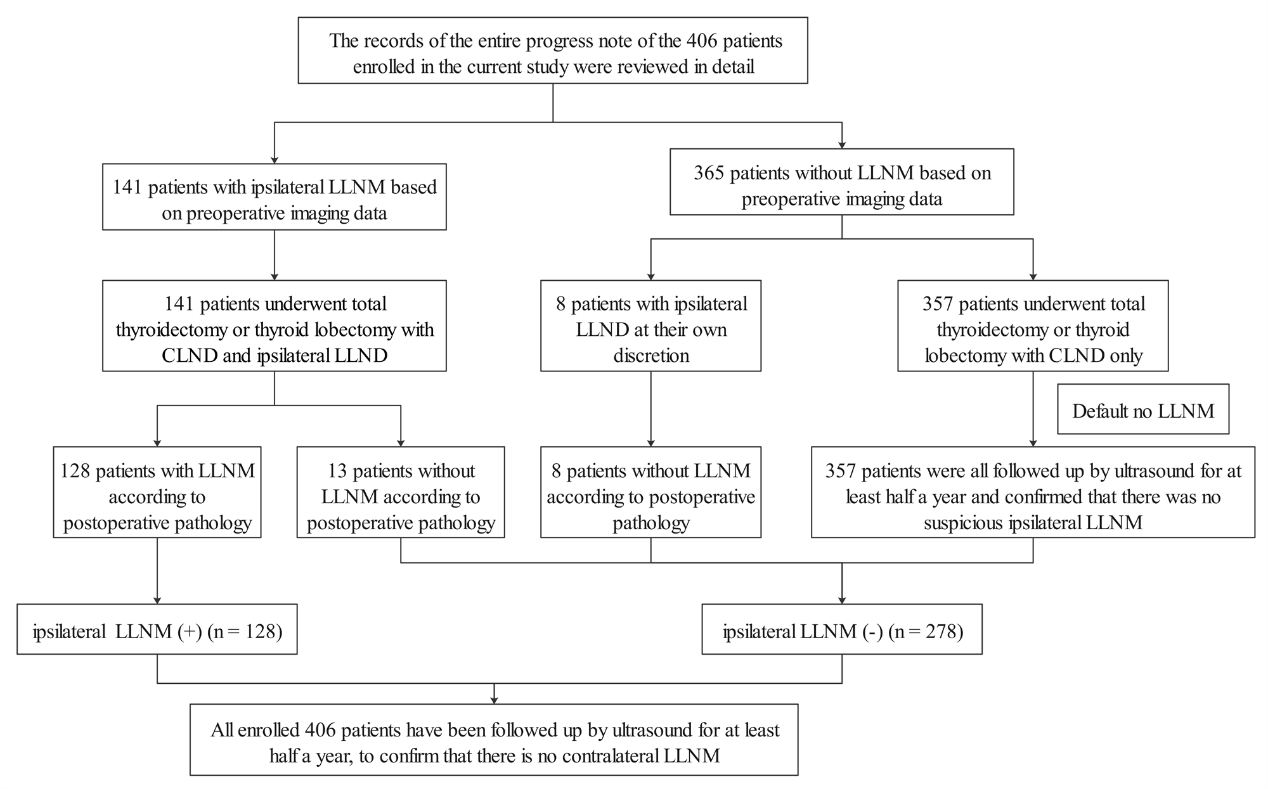


**Figure S1: Flowchart of the diagnosis of LLNM**

LLNM = lateral cervical lymph node metastasis, CLND = central cervical lymph node dissection, LLND = lateral cervical lymph node dissection

**
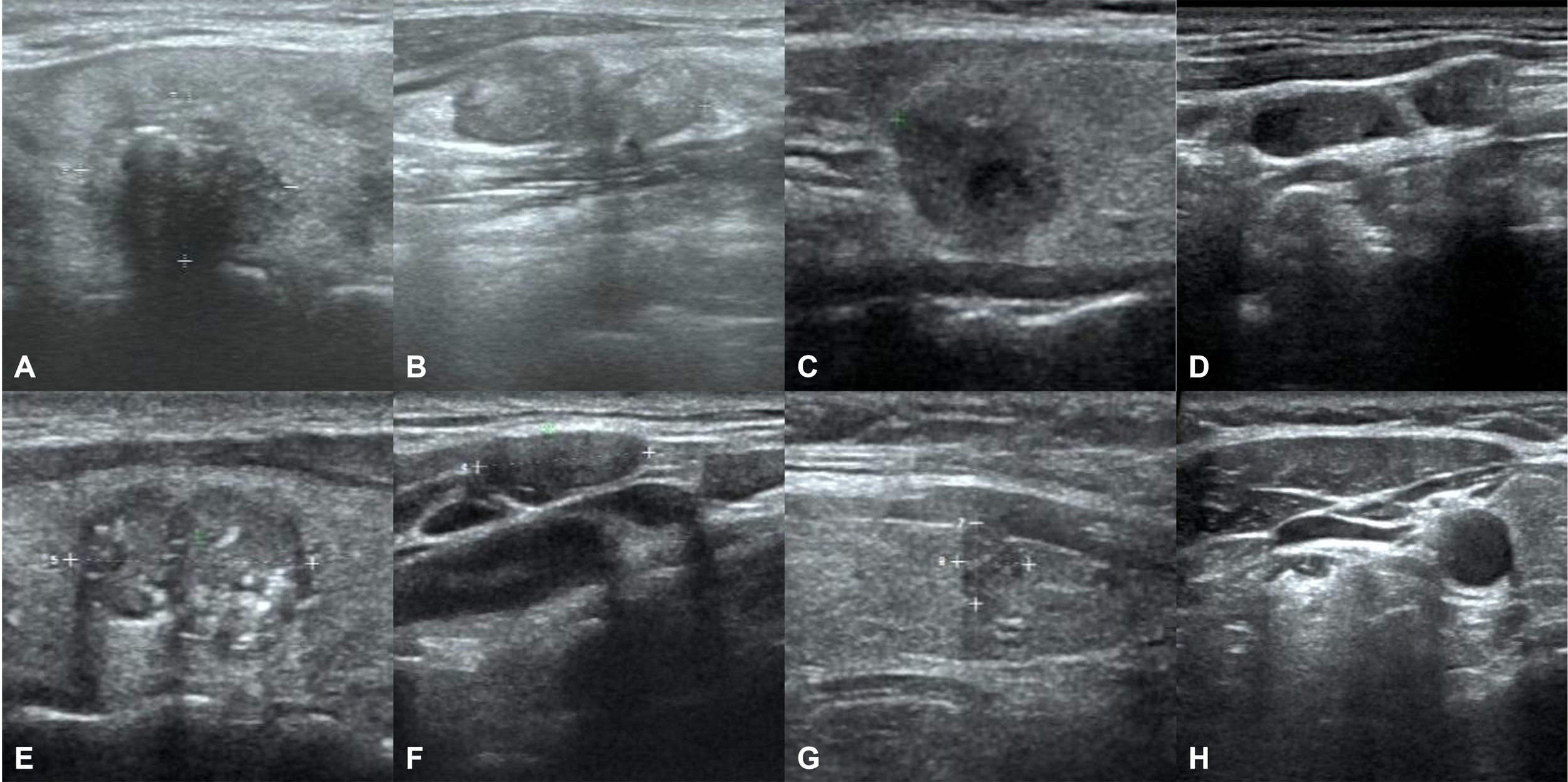
**

**Figure S2: Ultrasonic diagnostic standards for PTC and LNM.**

We give four cases to explain in detail the standards for ultrasound diagnosis of PTC and LNM. A, B, Hypoechoic nodules in the right lobe of the thyroid, with a diameter of about 1.4 cm, accompanied by microcalcification, and the boundary of the lesion is unclear. It is considered as malignant lesions (A). The hypoechoic nodule in the right level Ⅳ, about 1.8 cm in size, with unclear boundary, calcification, and the structure of the lymph node hilum is unclear, which is considered to be a metastatic disease (B). C, D, Hypoechoic nodules in the right lobe of the thyroid, with a diameter of about 1.3 cm, accompanied by microcalcification, taller-than-wide, and the boundary of the lesion is unclear. It is considered as malignant lesions (C). The hypoechoic nodule in the right level Ⅲ, about 1.0 cm in size, with unclear boundary, calcification, and the internal echo is uneven with cystic degeneration, which is considered to be a metastatic disease (D). E, F, Hypoechoic nodules in the left lobe of the thyroid, with a diameter of about 1.6 cm, accompanied by multiple divergence microcalcification, and the boundary of the lesion is unclear. It is considered as malignant lesions (E). The hypoechoic nodule in the left level Ⅱ, about 1.5 cm in size, with unclear boundary, and the internal echo is uneven with cystic degeneration, which is considered to be a suspected metastatic disease (F). G, H, Hypoechoic nodules in the left lobe of the thyroid, with a diameter of about 0.8 cm, accompanied by microcalcification, taller-than-wide, and the boundary of the lesion is unclear, which is considered as malignant lesions (G). No prominent enlarged lymph nodes in the left lateral cervical region (H).

PTC = papillary thyroid carcinoma, LNM = lymph node metastasis


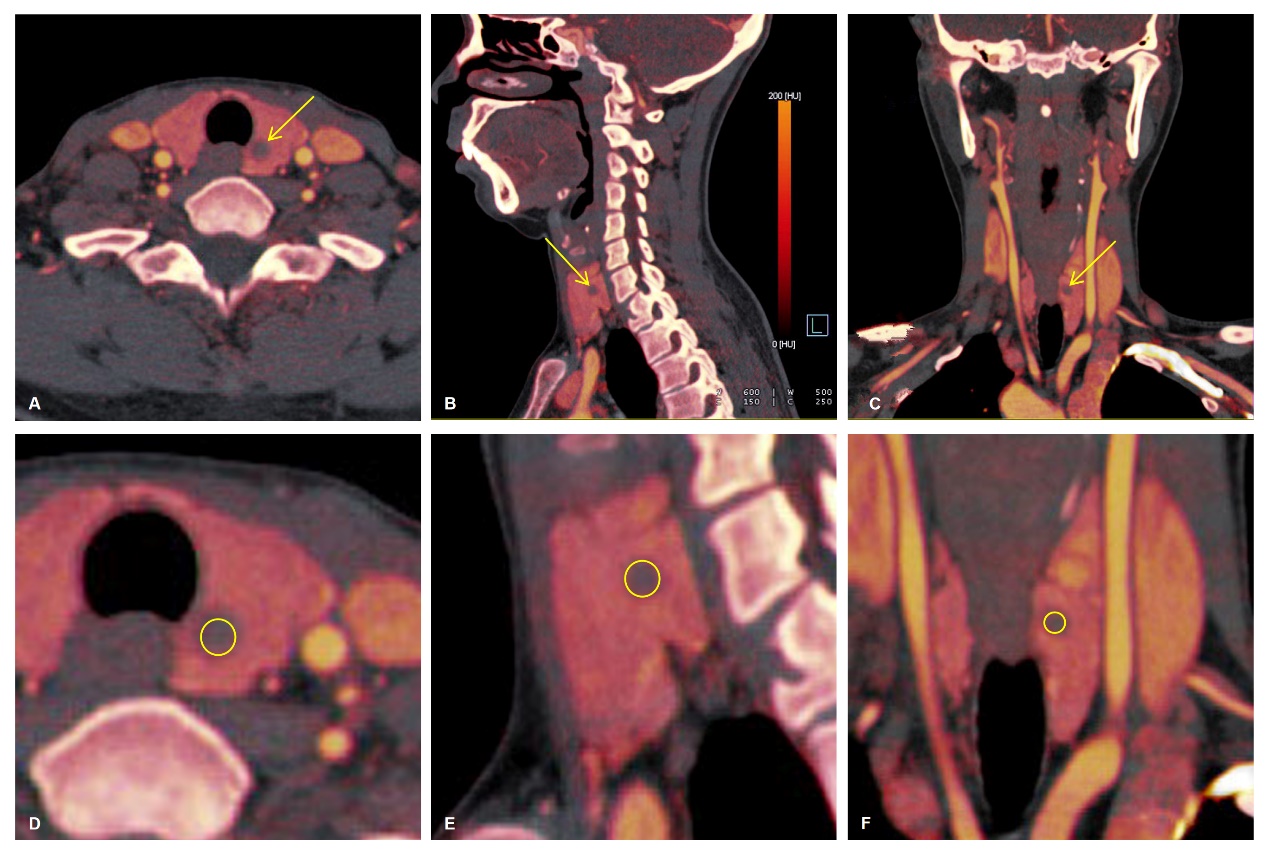


**Figure S3: The ROI of the primary lesion on the DECT images.**

A-C, The yellow arrow indicated a lesion located on the inferior dorsal pole of the left lobe of the thyroid in the axial, sagittal, and coronal iodine maps. D-F, An ovoid ROI (yellow circle; area, 78 mm^2^) was placed in the substantial part, including the entire lesion as large as possible, and avoiding peripheral fat, cystic, necrosis, and calcification.

ROI = region of interest, DECT = dual-energy computed tomography


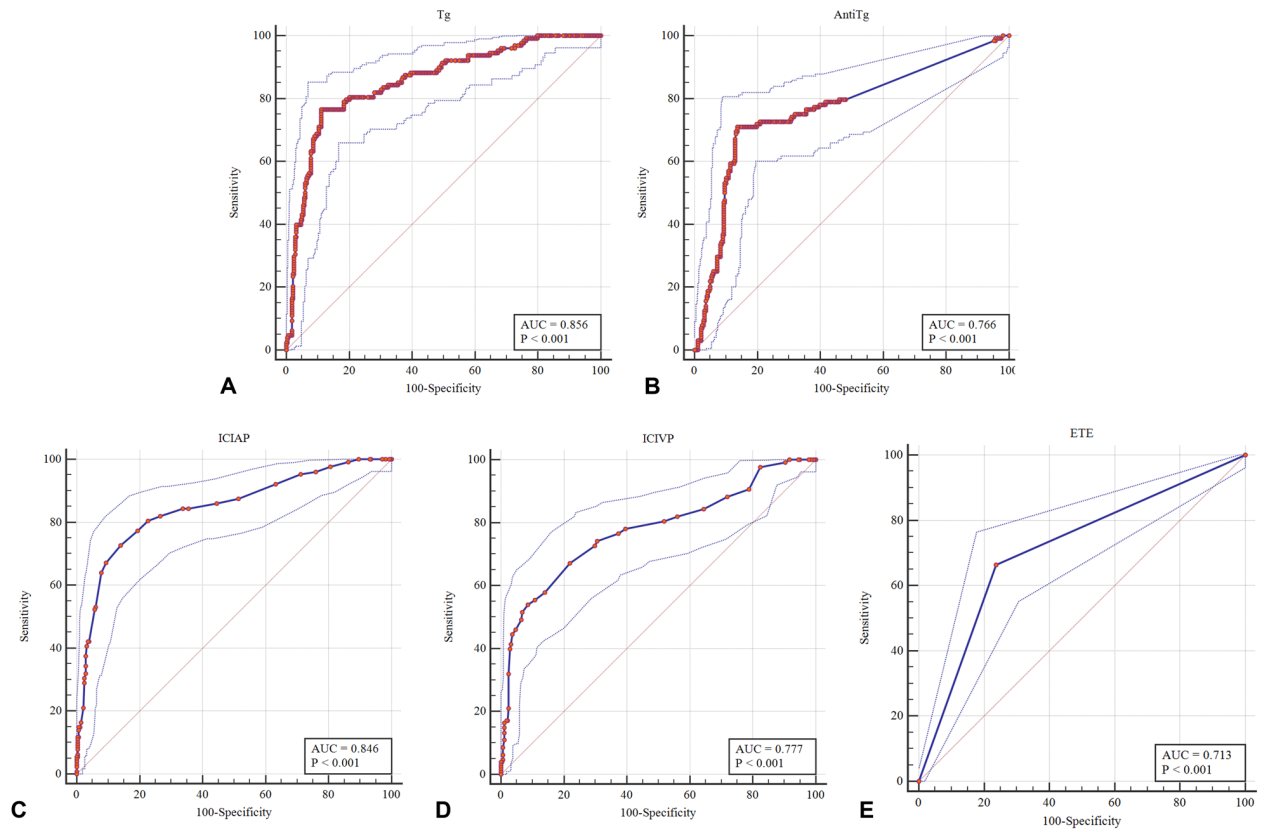


**Figure S4: ROC curves of DECT parameters and thyroid function indicators in patients with PTC.**

A, AUC, sensitivity, and specificity of Tg were 0.856, 76.56%, and 88.85%, respectively, with a cutoff value of 100.01 ng/ml. B, AUC, sensitivity, and specificity of Anti-Tg were 0.766, 71.09%, and 86.33%, respectively, with a cutoff value of 89.43 IU/ml. C, AUC, sensitivity, and specificity of IC IAP was 0.846, 72.66%, and 85.97%, respectively, with a cutoff value of 3.4 mg/ml. D, AUC, sensitivity, and specificity of IC IVP were 0.777, 53.91%, and 91.37%, respectively, with a cutoff value of 3.1 mg/ml. E, AUC, sensitivity, and specificity of ETE were 0.713, 66.41%, and 76.26%, respectively.

ROC = receiver operating characteristic, DECT = dual-energy computed tomography, PTC papillary thyroid carcinoma, AUC = the area under the curve, IC = iodine concentration, IAP = in the arterial phase, IVP = in the venous phase, ETE = extrathyroidal extension


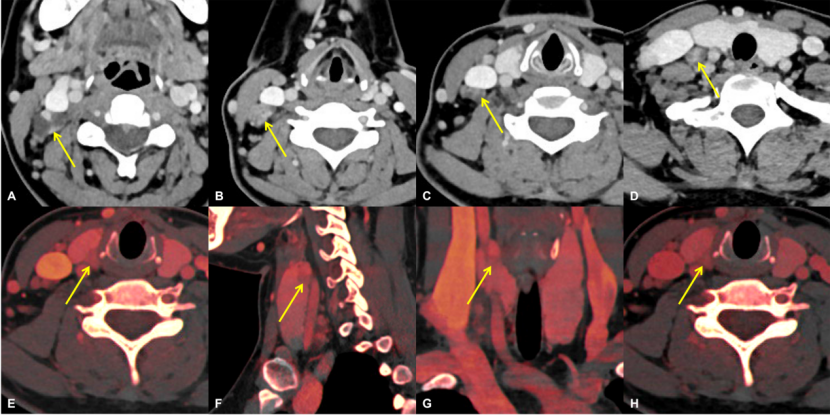


Figure S5: Neck contrast-enhanced CT indicated that multiple enlarged lymph nodes in right level Ⅱ-Ⅳ, which were considered metastatic lesions. (A) An enlarged lymph node located in right level Ⅱ, about 1.75 cm in diameter, heterogeneous enhancement with cystic change; (B) An enlarged lymph node located in right level Ⅲ, about 1.73 cm in diameter, uneven enhancement and with microcalcification at the edge; (C) An enlarged lymph node located in right level Ⅲ, about 1.26 cm in diameter, uneven enhancement with cystic change; (D) An enlarged lymph node located in right level Ⅳ, about 1.1 cm in diameter, with noticeable uneven enhancement. Postoperative pathology confirmed papillary carcinoma of the right lobe of the thyroid with a diameter of about 1.1cm, accompanied by lymph node metastasis in the right lateral cervical region. A review of dual-energy CT iodine maps showed that the iodine concentration of the primary lesion in the arterial phase (E-G) was 4.7 mg/mL and in the venous phase (H) was 4.5 mg/mL. And the primary lesion invaded the thyroid capsule. In other words, the preoperative dual-energy CT images of this patient suggested the possibility of LLNM to some extent.


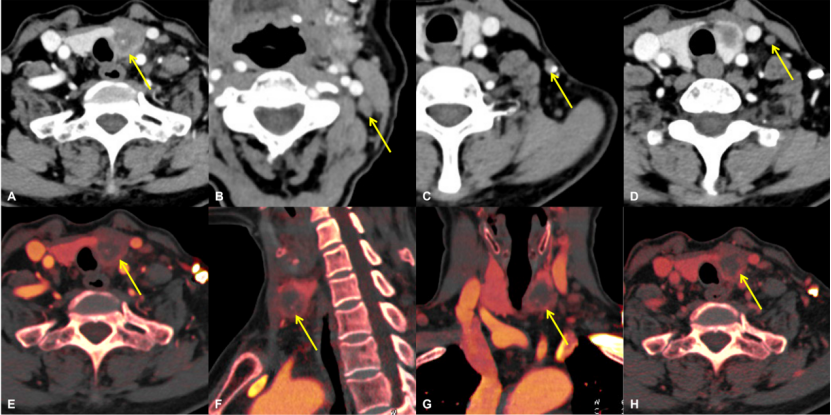


Figure S6: Neck contrast-enhanced CT indicated that multiple enlarged lymph nodes in left level Ⅱ, Ⅳ, and Ⅴ, which were considered metastatic lesions. (A) Primary lesion located in the left lobe of the thyroid, with cystic change and microcalcification; (B) An enlarged lymph node located in left level Ⅱ, about 1.1 cm in diameter; (C) A lymph node located in left level Ⅴ, about 0.8 cm in diameter, with microcalcification at the edge; (D) A round lymph node located in left level Ⅳ, about 0.6 cm in diameter. Postoperative pathology confirmed papillary carcinoma of the left lobe of the thyroid with a diameter of about 2.0 cm, and there was no metastatic lymph node in the left lateral cervical region. A review of dual-energy CT iodine maps showed that the iodine concentration of the primary lesion in the arterial phase (E-G) was 2.3 mg/mL and in the venous phase (H) was 2.0 mg/mL. In other words, the preoperative dual-energy CT images of this patient indicated that the possibility of lateral cervical lymph node metastasis was unlikely.


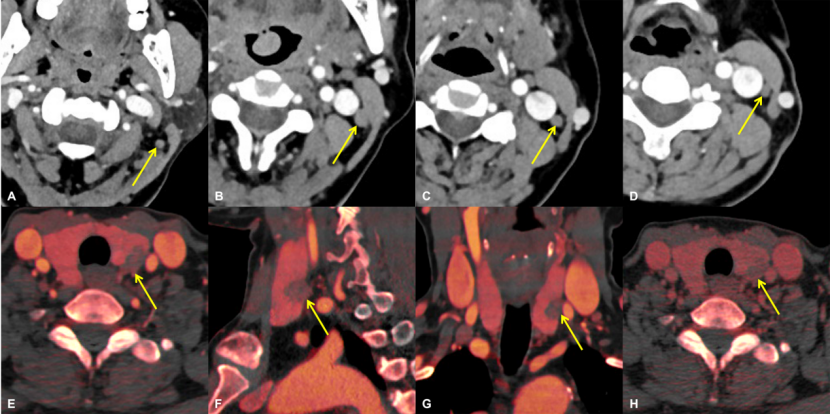


Figure S7: Neck contrast-enhanced CT indicated that no suspected metastatic lymph nodes in the lateral cervical region. (A-D) Four small lymph nodes located in left level Ⅱ, considered benign lesions. Postoperative pathology confirmed papillary carcinoma of the left lobe of the thyroid with a diameter of about 1.1 cm, and there was one metastatic lymph node in the left level Ⅱ. A review of dual-energy CT iodine maps showed that the iodine concentration of the primary lesion in the arterial phase (E-G) was 3.4 mg/mL and in the venous phase (H) was 3.2 mg/mL. In other words, the preoperative dual-energy CT images of this patient suggested the possibility of LLNM.

**References:**

1. Haugen BR, Alexander EK, Bible KC, Doherty GM, Mandel SJ, Nikiforov YE, et al. 2015 American Thyroid Association Management Guidelines for Adult Patients with Thyroid Nodules and Differentiated Thyroid Cancer. Thyroid : official journal of the American Thyroid Association. 2016.

2. Leenhardt L, Erdogan MF, Hegedus L, Mandel SJ, Paschke R, Rago T, et al. 2013 European thyroid association guidelines for cervical ultrasound scan and ultrasound-guided techniques in the postoperative management of patients with thyroid cancer. European thyroid journal. 2013;2(3):147-59.

3. Choi JS, Kim J, Kwak JY, Kim MJ, Chang HS, Kim EK. Preoperative staging of papillary thyroid carcinoma: comparison of ultrasound imaging and CT. AJR American journal of roentgenology. 2009;193(3):871-8.

4. Kim E, Park JS, Son K-R, Kim J-h, Jeon SJ, Na DG. Preoperative Diagnosis of Cervical Metastatic Lymph Nodes in Papillary Thyroid Carcinoma Comparison of Ultrasound, Computed Tomography. Thyroid research. 2008;18:411-8.

5. Liu X, Ouyang D, Li H, Zhang R, Lv Y, Yang A, et al. Papillary thyroid cancer: dual-energy spectral CT quantitative parameters for preoperative diagnosis of metastasis to the cervical lymph nodes. Radiology. 2015;275(1):167-76.
